# Hepatitis B Virus Subgenotype A1: Evolutionary Relationships between Brazilian, African and Asian Isolates

**DOI:** 10.1371/journal.pone.0105317

**Published:** 2014-08-14

**Authors:** Bárbara V. Lago, Francisco C. Mello, Anna Kramvis, Christian Niel, Selma A. Gomes

**Affiliations:** 1 Laboratory of Molecular Virology, Oswaldo Cruz Institute, FIOCRUZ, Rio de Janeiro, RJ, Brazil; 2 Hepatitis Virus Diversity Research Programme, Department of Internal Medicine, School of Clinical Medicine, Faculty of Health Sciences, University of the Witwatersrand, Johannesburg, South Africa; Academia Sinica & National Defense Medical Center, Taiwan

## Abstract

Brazil is a country of low hepatitis B virus (HBV) endemicity in which the genotype A of HBV (HBV/A) is the most prevalent. The complete nucleotide sequences of 26 HBV/A isolates, originating from eight Brazilian states, were determined. All were *adw2*. Twenty-three belonged to subgenotype A1 and three to A2. By phylogenetic analysis, it was shown that all the 23 HBV/A1 isolates clustered together with isolates from Bangladesh, India, Japan, Nepal, the Philippines and United Arab Emirates, but not with those of Congo, Kenya, Malawi, Rwanda, South Africa, Tanzania, Uganda and Zimbabwe. Four amino acid residues in the polymerase (His138 in the terminal protein domain, Pro18 and His90 in the spacer, and Ser109 in the reverse transcriptase), and one (Phe17) in the precore region, predominated in Latin American and Asian HBV/A1 isolates, but were rarely encountered in African isolates, with the exception of those from Somalia. Specific variations of two adjacent amino acids in the C-terminal domain of the HBx protein, namely Ala146 and Pro147, were found in all the Brazilian, but rarely in the other HBV/A1 isolates. By Bayesian analysis, the existence of an ‘Asian-American’ clade within subgenotype A1 was supported by a posterior probability value of 0.996. The close relatedness of the Brazilian, Asian and Somalian isolates suggests that the HBV/A1 strains predominant in Brazil did not originate from the five million slaves who were imported from Central and Western Africa from 1551 to 1840, but rather from the 300–400,000 captives forcibly removed from southeast Africa at the middle of the 19^th^ century.

## Introduction

Worldwide, more than 240 million people are chronically infected with hepatitis B virus (HBV) [1]. HBV prevalence, measured by the presence of hepatitis B surface antigen (HBsAg) in the serum, varies from <1% to >10% depending on the geographic region, with the highest prevalence in sub-Saharan Africa and southeast Asia. Based on a genomic sequence divergence >7.5% over the entire DNA genome, HBV isolates have been classified into eight genotypes (HBV/A to H) [2]. Additionally, two genotypes (I and J) have been proposed [3], [4]. Genotypes A–D and F are further divided into subgenotypes that show a sequence divergence of about 4% between them [2]. HBV genotypes and subgenotypes have distinct geographic distribution and may be responsible for differences in the natural history and clinical outcome of the infection [5]–[8].

Originally, genotype A was classified into two subgenotypes A1 and A2 [9]–[11]. Five additional subgenotypes, namely A3 [12], [13], A4 and A5 [14], A6 [15], and A7 [16] have been identified in African individuals. Recently, an alternative classification was proposed in which the subgenotypes A3, A4, A5 and A7 are classified into the quasi-subgenotype A3, and subgenotype A6 renamed A4 [17], [18]. Subgenotypes A1, A4 and quasi-subgenotype A3 are found mainly in Africa, whereas A2 prevails outside Africa. It was earlier proposed that genotype A has an African origin, with subgenotype A1 prevailing in sub-Saharan Africa and the Indian subcontinent, and A2, predominant in Europe and North America, having spread more than 500 years ago from Africa to Asia and Europe, respectively [12]. The African origin of subgenotype A1 was recently shown by phylogeographic analysis [19].

Brazil is a country of low HBV endemicity, with an estimated HBsAg prevalence of 0.25–0.50% in the general population [20], although higher prevalence rates (1.6–3.3%) have been found in the Amazon Basin [21], [22]. Countrywide, genotype A is the most common, followed by genotypes D and F [23]. Genotype A is predominant in the northern, northeast and southeast regions [24] which together, account for 79% of the countrýs population. Within genotype A, subgenotype A1 has been found at a frequency about ten times higher than A2 [24]–[27]. It has been suggested that the circulation of HBV genotypes A, D, and F reflects the ancestral descent of the Brazilian population [23]. The origin of the Brazilian population can be traced from three main sources, namely the Amerindians, who are mainly HBV/F carriers [28], [29], the European colonizers, responsible for the entry of genotypes A2 and D into Latin America [30], [31], and the African slaves, who would have been carriers of HBV/A1 when they arrived in Brazil between the 16^th^ and the 19^th^ century. The proposal that subgenotype A1 was introduced into Brazil by the arrival of slaves [23], [24] has been reinforced by the observations that (i) HBV/A1 is almost the exclusive subgenotype found in semi-isolated Afro-descendant communities [32]–, and (ii) Blacks and Mulattos (mixed descent) are more frequently infected with subgenotype A1 than caucasians, whereas the contrary is true for genotype D [30].

Geographically, subgenotype A1 is found in three ecoregions: Africotropic, Indo-Malay and Neotropic. When the complete genomes of HBV strains from Africa were compared to those outside Africa, with the exception of the Somalian strains, the majority of African sequences clustered separately from the Asian/American sequences, thus suggesting that subgenotype A1 was dispersed outside Africa by the slave and trade routes of the 15^th^ to 19^th^ centuries [19]. Brazil is the country that received more than five million of the 12 million slaves, forcibly removed from Africa, and was the last country to ban the Atlantic slave trade in the second half of the 19^th^ century. The aim of this study was to use phylogenetic and phylogeographic analyses of complete sequences of HBV in order to shed light on the origins of subgenotype A1 in Brazil.

## Materials and Methods

### Ethics statement

The human blood samples from the present study were collected in several hepatitis reference centers located in different regions of Brazil. The study was approved by the Brazilian Ethics Committee for Medical Research (Comissão Nacional de Ética em Pesquisa - CONEP registration number 9604/2004). All the participants knew to be HBV chronic carriers and gave their written consent to participate to the study. Consent forms were recorded in the reference centers and kept separately from questionnaires. The names of the patients could not be linked to any study data collected.

### HBV samples

The 26 HBV isolates genetically characterized in this study were derived from blood donors (n = 13), patients with acute (n = 5) or chronic (n = 5) hepatitis B, and men who have sex with men (n = 3). The samples, which originated from eight Brazilian states, namely Amapá (n = 2), Amazonas (n = 3), Goiás (n = 1), Mato Grosso do Sul (n = 3), Pernambuco (n = 6), Rio de Janeiro (n = 6), Santa Catarina (n = 2) and São Paulo (n = 3), had been previously shown to belong to genotype A by polymerase chain reaction-restriction fragment length polymorphism (PCR-RFLP) analysis [23], [24].

### Viral DNA extraction and PCR amplification

HBV DNA was extracted from 200 µl of serum sample using *High Pure Viral Nucleic Acid kit* (Roche Diagnostics, Mannheim, Germany) according to the manufacturer’s instructions. The HBV whole genome was PCR amplified according to a previously published method using oligonucleotide primers P1 and P2 [35]. These primers have been designed at the 5′ and 3′ ends of the viral minus-strand DNA that exhibit a 9-nucleotide-long terminal redundancy.

### Nucleotide sequencing

After 0.8% agarose gel electrophoresis, PCR products were purified by using the *Wizard SV Gel and PCR Clean-Up System* (Promega, Fitchburg, WI). Nucleotide sequences of full-length HBV genomes were determined by direct sequencing using the *BigDye Terminator v3.1 Cycle Sequencing kit* (Applied Biosystems, Foster City, CA) and a set of eight sense and eight antisense specific primers distributed along the HBV genome [36]. Sequencing reactions were analyzed on an ABI 3730 automated sequencer (Applied Biosystems). The nucleotide sequences have been deposited in the GenBank database (accession numbers KJ854685 to KJ854710).

### Phylogenetic analysis

Multiple sequence alignment was performed by using Clustal X program with 151 HBV/A complete nucleotide sequences including the 26 sequences determined in this study, 111 HBV/A1 sequences available in the GenBank database originating from 20 countries (Argentina [n = 3], Bangladesh [n = 2], Colombia [n = 4], Congo [n = 1], France [n = 1], Haiti [n = 34], India [n = 7], Japan [n = 2], Kenya [n = 4], Malawi [n = 2], Martinique [n = 6], Nepal [n = 2], the Philippines [n = 7], Rwanda [n = 7], Somalia [n = 7], South Africa [n = 18], Tanzania [n = 1], Uganda [n = 1], United Arab Emirates [n = 1] and Zimbabwe [n = 1]), and 14 A2–A4 previously determined sequences which were used as an outgroup. The list of the GenBank accession numbers of the sequences, along with their subgenotypes and countries of origin, is shown in Table S1. Phylogenetic analysis was carried out using the maximum likelihood method (bootstrap resampling test with 1,000 replicates) in MEGA version 5.1 software.

### Bayesian Inference of migration events

In order to investigate the HBV/A1 migratory patterns and starting points of diversification, Bayesian phylogenetic analysis was conducted using Markov Chain Monte Carlo (MCMC) simulation implemented in BEAST v1.7.4 [37]. Differently from the other experiments, not all HBV/A1 isolates from Brazil (n = 23), Haiti (n = 34) and South Africa (n = 18) were included in the phylogeographic analysis. This was done to avoid over-representation of these countries, which might bias the ancestral location estimates. When two or more isolates from one of these countries shared >99% homology to each other, only one sequence was included in the analysis. The remaining HBV/A1 isolates (n = 101) were classified into eight geographic regions to establish the more plausible routes of dissemination: Antilles (Haiti, Martinique), Hispanic America (Argentina, Colombia), Brazil, Asia (Bangladesh, India, Japan, Nepal, the Philippines, United Arab Emirates), Europe (France), Somalia, South Africa, and other Sub-Saharan countries (Congo, Kenya, Malawi, Rwanda, Tanzania, Uganda, Zimbabwe). Bayesian trees were calibrated using six different, previously published evolutionary rates between 2.2×10^−6^ and 7.7×10^−4^ substitutions/site/year (s/s/y) [38]–[42]. The time of the most recent common ancestor (tMRCA) of internal nodes was estimated under an uncorrelated lognormal relaxed molecular clock model. MCMC was run for 60×10^6^ generations using a general time reversible with gamma distributed rate heterogeneity and estimated proportion of invariable sites (GTR+G+I) substitution model (BEAST version 1.7.4), with a burn-in of 15%. The convergence of the Markov chain (estimated sum of squares >200) was assessed using Tracer version 1.5. The consensus tree was estimated by the TreeAnnotator programme v1.6.1. [37].

## Results and Discussion

### Geographic distribution of HBV subgenotypes A1 and A2 in brazil

The complete genomes of 26 HBV/A isolates, originating from eight Brazilian states, were successfully amplified and sequenced. To our knowledge, these represented the first Brazilian HBV/A isolates to be fully sequenced. All isolates had a genome size of 3,221 nucleotides. Deduced amino acid sequences of the small S protein of all isolates showed Lys, Pro and Lys residues at positions 122, 127 and 160, respectively, corresponding to the *adw2* serological subtype. By phylogenetic analysis (see below), it was found that 23 isolates belonged to subgenotype A1 and three to subgenotype A2. In six states (Amapá, Amazonas, Goiás, Mato Grosso, Pernambuco and Rio de Janeiro), all the isolates under study belonged to subgenotype A1. The three A2 isolates were from the southern states, Santa Catarina (two samples) and São Paulo (one sample). São Paulo was the only state where both subgenotypes A1 and A2 were identified.

**Figure 1:**
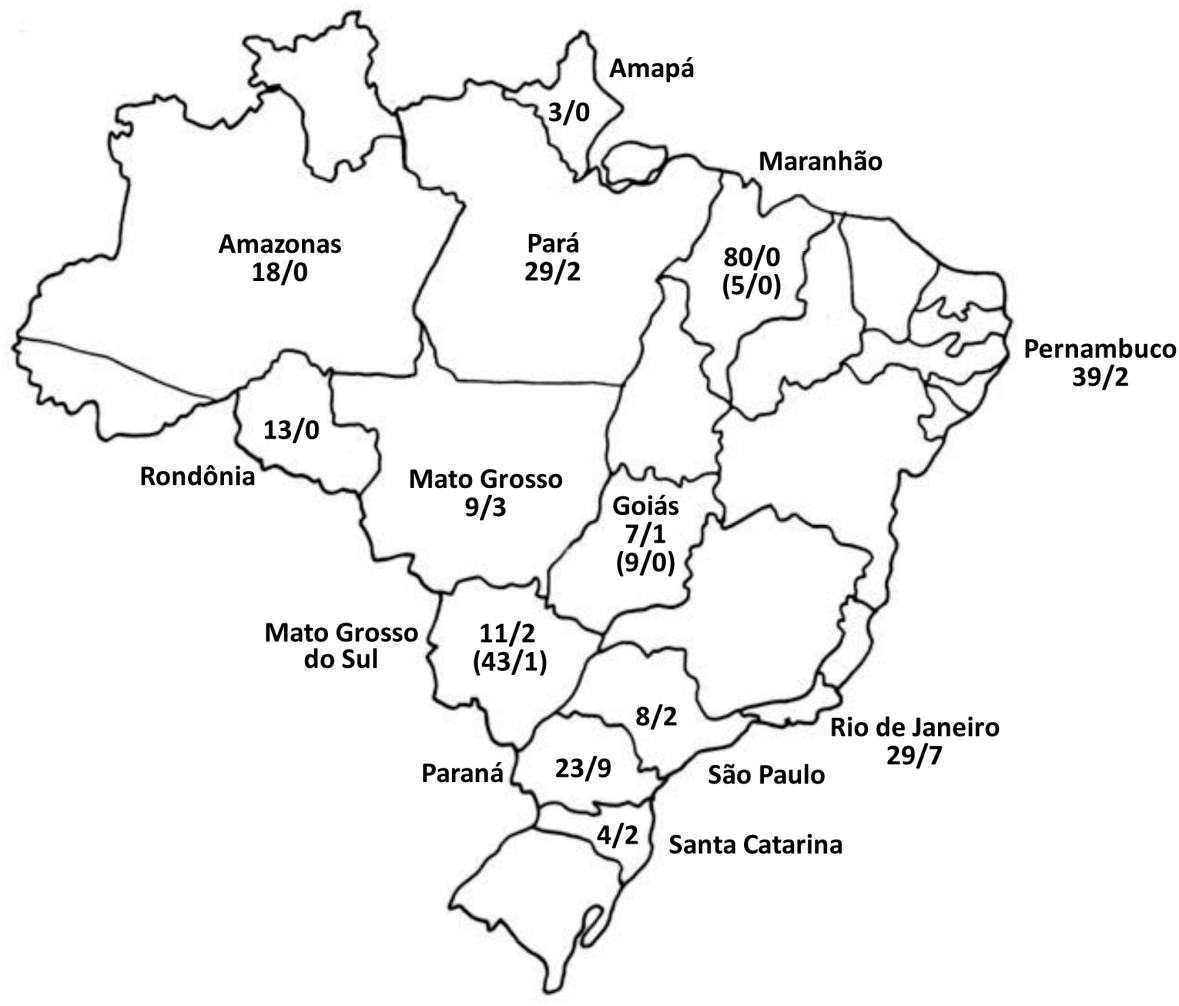
Geographical distribution of HBV/A1 and A2 isolates in Brazil, including the 26 isolates characterized in this work. Numbers indicate the ratios A1/A2. Numbers in parentheses refer to semi-isolated Afro-Brazilian communities which were established by the slave escapees during the 17^th^ and 18^th^ centuries. Data compiled from [24]–[27], [30], [43], [44].

Based on PCR-RFLP and partial nucleotide sequencing, subgenotypes A1 and A2 have previously been identified in Brazil [24]–[27], [30], [43], [44]. No HBV isolate belonging to quasi-subgenotype A3 and subgenotype A4 has been identified in Brazil. Figure 1 shows the geographic distribution of all the HBV/A isolates identified in Brazil to date, including the 26 characterized in this study. Data were available for 13 of the 26 Brazilian states. Although in all states, the frequency of subgenotype A1 was higher than that of A2, the ratio of A1:A2 was >14 in the northern states of Amapá, Amazonas, Maranhão, Pará, Pernambuco and Rondônia, whereas it was <5 in the southern states of Paraná, Rio de Janeiro, Santa Catarina and São Paulo (Fig. 1).

The proportion of European descendants, mainly Portuguese, Italian, Spanish, German and Polish, is higher in southern Brazil compared to other parts of the country, where Black and Mulatto people are generally in the majority. Considering the geographic distribution of the two subgenotypes (A1 in Africa and A2 in Europe), the prevalence of A2 in the southern Brazil correlated with the European origin of the population. On the other hand, almost all HBV isolates were shown to belong to subgenotype A1 in three Afro-descendant communities living in relative isolation since the slavery period in the states of Maranhão [32], Goiás [33] and Mato Grosso do Sul [34] (data in parentheses on Fig. 1).

### All HBV/A1 brazilian isolates cluster in the ‘Asian-American’ clade

Two distinct clades have been recognized into subgenotype A1. One clade is mainly ‘African’ and includes isolates from southern, eastern and central Africa, while the other, comprising isolates from Asia and Somalia, has been called ‘Asian’ [45]–[47] or ‘Afro-Asian’ [48]. Recently, the term ‘Asian-American’ has also been used to design this latter clade, based on phylogenetic analyses that included HBV/A1 isolates from Caribbean islands and a restricted number of South American strains [19], [31]. The ‘Asian-American’ clade would have originated in the nineteenth century when African slaves were exported from Mozambique to Central and South America, the islands of the Indian Ocean, and India. As Brazil has been the main importer of slaves for a long period of time and the last nation to outlaw the slave trade, it was essential to perform genetic distance and phylogenetic analyses with a sufficient number of Brazilian HBV/A1 isolates to validate or not this hypothesis.

Table 1 shows the intragroup and intergroup mean divergences between HBV/A1 isolates from different geographical regions. Even though the 23 Brazilian strains were sampled from geographically distinct regions of the country, their mean intragroup divergence was relatively low, 1.14 (95% CI 0.94–1.34). The other intragroup divergences were included between 1.14 and 2.07, similar to previous observations [19], [49]. The mean intergroup divergence between Brazilian and non-Brazilian isolates ranged from 1.40 to 2.28, with the lowest divergence from isolates from the Antilles and the highest with African isolates. Intergroup mean divergences between Brazilian and Asian (1.69) or Somalian (1.60) isolates were markedly lower than those observed between Brazilian strains and isolates from South Africa (2.24) or other sub-Saharan countries (2.28), although the upper confidence limits of the Brazilian-Asian (1.93) and Brazilian-Somalian (1.89) divergences were slightly higher than the lower confidence limit (1.87) of the Brazilian-sub-Saharan divergence (small overlap; see Table 1).

**Table 1:** Intragroup and intergroup mean divergences between HBV/A1 isolates from different geographic regions.

Continent	Country/region (number of isolates)	(Intragroup divergence (95% CI*)	Divergence with Brazilian isolates (95% CI)
Americas	Brazil (23)	1.14 (0.94–1.34)	-
	Antilles (40)	1.14 (0.96–1.32)	1.40 (1.16–1.64)
	Hispanic America (7)	1.91 (1.58–2.24)	1.91 (1.58–2.24)
Asia	Various (21)	1.71 (1.49–1.93)	1.69 (1.45–1.93)
Africa	Somalia (7)	1.36 (0.97–1.65)	1.60 (1.31–1.89)
	South Africa (18)	2.07 (1.80–2.34)	2.24 (1.89–2.59)
	Other sub-Saharan (17)	1.57 (1.33–1.81)	2.28 (1.87–2.69)
Europe	France (1)	-	1.71 (1.26–2.16)

*CI, confidence interval.

**Figure 2:**
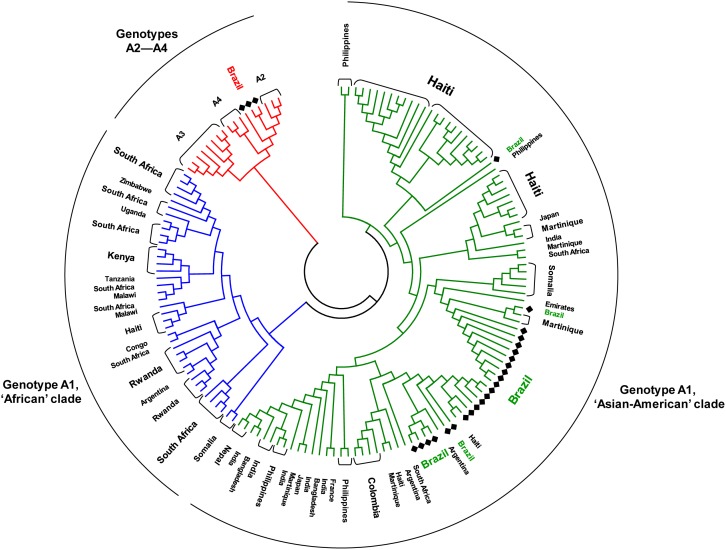
Phylogenetic analysis based on HBV complete nucleotide sequences. The phylogenetic tree, performed by using the maximum likelihood method, incorporates 134 HBV/A1 isolates, including the 23 Brazilian isolates sequenced in this study. Subgenotype A1 strains separated into two clades, ‘African’ (blue) and ‘Asian-American’ (green). In addition, sequences of A2, quasi-subgenotype A3 and A4 samples were included as an outgroup (red), which allowed three Brazilian samples to be classified as subgenotype A2. The list of the GenBank accession numbers of the isolates with their subgenotypes and countries of origin is shown in Table S1.

Figure 2 shows a phylogenetic tree obtained by using the maximum-likelihood method, based on the complete nucleotide sequences of HBV/A1 isolates available in GenBank, the sequences from this work, and 14 sequences of A2–A4 isolates which were used as an outgroup. Markedly, all HBV/A1 isolates from Brazil (n = 23) and the Asian countries (Bangladesh, n = 2; India, n = 7; Japan, n = 2; Nepal, n = 2; Philippines, n = 7; United Arab Emirates, n = 1) clustered together, which corroborated the hypothesis of the ‘Asian-American’ clade (represented in green on Fig. 2). All the Colombian (n = 4), French (n = 1) and Martinican (n = 6) isolates, as well as 2/3 Argentinian, 31/34 Haitian and 5/7 Somalian isolates, were also present in this clade. On the other hand, all the isolates from seven sub-Saharan countries, namely Congo (n = 1), Kenya (n = 4), Malawi (n = 2), Rwanda (n = 7), Tanzania (n = 1), Uganda (n = 1) and Zimbabwe (n = 1), as well as 16/18 South African isolates, clustered into the other, ‘African’ clade, represented in blue.

Three Brazilian isolates clustered together in a branch containing A2–A4 isolates from other countries (Fig. 2). An analysis (not shown) using the blast algorithm (http://blast.ncbi.nlm.nih.gov/Blast.cgi) demonstrated that the three Brazilian isolates showed >99% sequence homology with HBV/A2 isolates from Russia, Poland and Belgium, and 94.8–96.1% homology with three A4 (formerly A6) isolates described previously [15], allowing their unambiguous classification into subgenotype A2.

### Amino acid variability among HBV/A1 isolates from different geographic regions

The deduced amino acid sequences of the polymerase, precore-core, surface antigen and X protein (HBx) of the 134 HBV/A1 isolates used to construct the phylogenetic tree (Fig. 2) were compared. The frequency of determined amino acid residues varied according to the geographical origin of the isolates. Table 2 shows the 10 positions where the most notable variations of frequency were observed. Four amino acids in the polymerase (His138 in the terminal protein domain, Pro18 and His90 in the spacer, and Ser109 in the reverse transcriptase domain), and one (Phe17) in the precore region, were found to be largely predominant (71–100%) in Latin America (Antilles, Brazil and Hispanic America) and Asia, but rarely encountered in Africa, except in Somalia. Specific variations of two adjacent amino acids in the C-terminal domain of the HBx protein, namely Ala146 and Pro147, were found in all the Brazilian, but rarely in the other HBV/A1 isolates. However, these two residues are part of the consensus amino acid sequences of (sub)genotypes A2 and B–H [50].

**Table 2:** Frequencies of amino acid residues in the proteins of HBV/A1 isolates: Correlation with the geographic region.

		Polymerase						Precore	HBx		
Continent	Country/region	tp^a^ 91	tp 138	spacer 18	spacer 90	rt^b^ 109	rt 153	17	32	146	147
America	Brazil	Ala (23/23)	His (23/23)	Pro (20/23)	His (22/23)	Ser (23/23)	Arg (13/23)	Phe (22/22)	Arg (15/22)	Ala (20/20)	Pro (21/21)
	Antilles	Ser (30/39)	His (30/40)	Pro (33/38)	His (37/40)	Ser (37/40)	Trp (36/40)	Phe (35/40)	Arg (27/40)	Ser (36/38)	Ser (38/40)
	Hispanic America	Ala (6/7)	His (5/7)	Pro (6/7)	His (6/7)	Ser (6/7)	Trp (5/7)	Phe (6/7)	Gly (6/7)	Ser (7/7)	Ser (7/7)
Asia	Various^c^	Ala (14/21)	His (17/21)	Pro (19/21)	His (21/21)	Ser (21/21)	Trp (19/21)	Phe (19/21)	Arg (15/21)	Ser (17/20)	Ser (18/21)
Africa	Somalia	Ile (4/7)	Gln (4/7)	Pro (5/7)	His (6/7)	Ser (6/7)	Trp (7/7)	Phe (6/7)	Gly (6/7)	Ser (6/7)	Ser (6/7)
	South Africa	Ile (16/18)	Gln (16/18)	Ser (16/18)	Tyr (14/18)	Pro (14/18)	Trp (18/18)	Val (14/18)	Gly (15/18)	Ser (14/18)	Ser (12/18)
	Others^d^	Ile (17/17)	Gln (16/17)	Ser (17/17)	Tyr (17/17)	Pro (11/17)	Trp (17/17)	Val (16/17)	Gly (17/17)	Ser (12/16)	Ser (15/17)
Europe	France	Ile (1/1)	Gln (1/1)	Pro (1/1)	His (1/1)	Ser (1/1)	Trp (1/1)	Phe (1/1)	Arg (1/1)	Ser (1/1)	Ser (1/1)

a tp, terminal protein domain;

b rt, reverse transcriptase domain;

c Bangladesh, India, Japan, Nepal, Philippines, United Arab Emirates;

d Congo, Kenya, Malawi, Rwanda, Tanzania, Uganda, Zimbabwe.

The different HBV genotypes and subgenotypes display specific amino acids and genome lengths variations. In the last years, the role of specific amino acid variations in the different HBV proteins has been extensively studied These variations could affect virus antigenicity, HBeAg expression, replication rate, speed of disease progression, reliability of diagnostic methods and the success of antiviral therapy and immunization [51], [52]. However, a review of the literature did not reveal a clinical significance or a shift of the replication rate for any of the 10 geographic region-specific mutations listed in Table 2.

### Phylogeographic analysis

**Figure 3:**
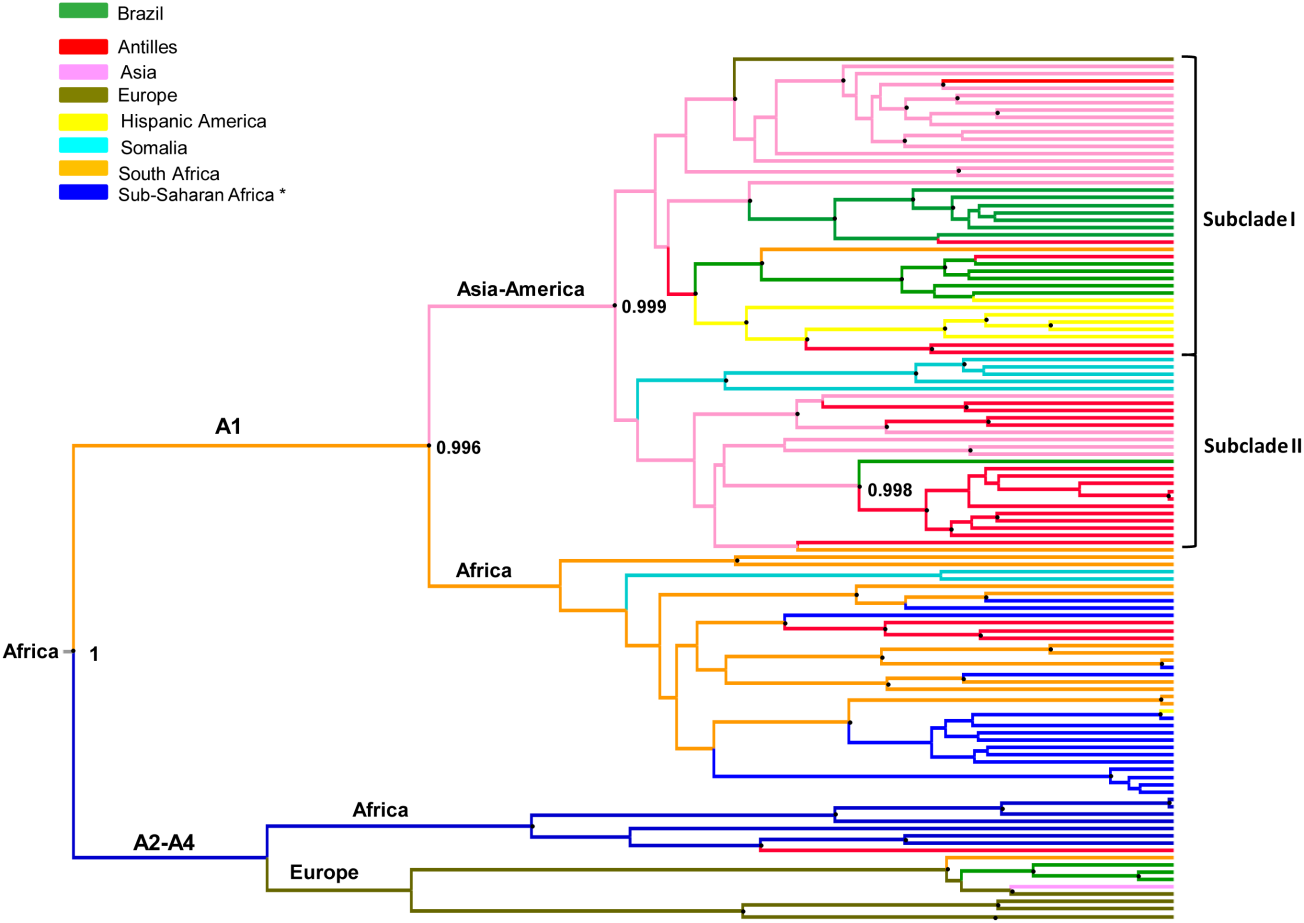
Bayesian maximum clade credibility tree of HBV/A full length nucleotide sequences. A1 and A2–A4 represent HBV subgenotypes within genotype A. The numbers on the internal nodes represent posterior probabilities. All nodes marked with a dot showed posterior probability >0.95. The branch lengths are proportional to length of time. The names of the continents on the branches indicate the most plausible routes of dissemination. *Sub-Saharan Africa refers to Congo, Kenya, Malawi, Rwanda, Tanzania, Uganda and Zimbabwe.

Bayesian analysis was conducted to investigate the migratory patterns and starting points of diversification of HBV/A1. On the maximum clade credibility tree shown on Figure 3, HBV most probable dissemination routes are represented by different colours, corresponding to different parts of the world. It was confirmed that genotype A originated from Africa, as previously proposed [12]. Within subgenotype A1, the separation between the ‘African’ and ‘Asian-American’ clades was corroborated with a posterior probability value of 0.996. ‘African’ clade included all A1 isolates from sub-Saharan Africa (Congo, Kenya, Malawi, Rwanda, Tanzania, Uganda and Zimbabwe), all but one sequence from South Africa, as well as a few isolates from Somalia and the Antilles (Haiti). All isolates from Asia (Bangladesh, India, Japan, Nepal, Philippines, United Arab Emirates), most of the isolates from Hispanic America, the Antilles and Somalia, as well as one sequence from South Africa, were located into the ‘Asian-American’ clade. Similarly to what was found by phylogenetic analysis (Fig. 2), all HBV/A1 Brazilian isolates clustered in the ‘Asian-American’ clade by Bayesian inference. The ‘Asian-American’ clade diverged from the ‘African’ clade moving toward Asia (pink colour on Fig. 3) and, from then, was divided into two subclades (I and II) with a posterior probability of 0.999. Subclade I acted as the source of infection for Asia, Brazil and the Hispanic America, with a single isolate going in the opposite direction, from Brazil to South Africa. Subclade II was composed of a monophyletic group formed by Somalian isolates plus a large group containing isolates from Asia (The Philippines), heading for the Antilles (Martinique and Haiti). One Brazilian isolate clustered together in the latter group, close to the Haitian isolates, with a posterior probability of 0.998.

The tree represented on Figure 3 was constructed using the value of 4.6×10^−5 ^s/s/y as the evolutionary rate [40]. Five additional trees were constructed by using previously published evolutionary rates [38], [39], [41], [42] varying from 2.2×10^−6^ to 7.7×10^−4^. All of them showed a very similar topology (not shown). The only notable difference was that Somalia substituted Asia as the origin of the ‘Asian-American’ branch in the tree constructed by using an evolutionary rate of 1.4×10^−5 ^s/s/y [39]. Such a common ancestry of Somalian, Asian and Latin American HBV/A1 isolates might be explained by the Arab slave trade which was the practice of slavery in the Arab world, mainly in Western Asia, North Africa, Southeast Africa, the Horn of Africa and certain parts of Europe during the era of the Arab conquests [19].

It is historically known that West Central Africa (Angola) and the Bight of Benin, in this order, were the major suppliers of slave labor to Brazil (4–5 million people; 16–19^th^ century) [53]. However, an estimated number of 300–400,000 captives were brought from southeast Africa (Mozambique) between 1837 and 1856, circumventing the laws and treaties that prohibited the transatlantic slave trade [54]. A previous phylogeographic investigation of the Afro-descendant population of São Paulo, based on haplogroups of the Y chromosome and mitochondrial DNA, has reported that 87.6% of black Brazilians are descendants of people originated from central and western Africa, while 12.3% descended from southeast Africa [55]. Despite this low proportion, our findings using phylogeographic analysis of complete HBV genomes intimate that southeast Africa was the main source of HBV entry in Brazil, further corroborating the phylogeographic analysis done using only the S region and a limited number of Brazilian strains [19]. Studying the genetics of individuals infected with HBV/A1 could help in determining the origin of the human population infected by this genotype. Such a study, however, could have limitations due to (i) the time elapsed since the arrival in Brazil of slaves from the East African coast (160–180 years, 5–6 human generations) and (ii) the fact that the vast majority of these slaves were mixed immediately with the slaves from West Africa arrived before them, and mixed later with the general population.

Another possibility would be that HBV/A1 was brought from India to Brazil by Portuguese and Brazilian traders. However, this hypothesis does not explain the predominance of HBV/A1 in the Brazilian Black and Mulatto populations [30] and Afro-descendant communities [32]–[34].

It is noteworthy that genotype E, which is presently the predominant genotype in Angola [56], [57], is rarely found in South America. The absence of the genotype E in Angola at the time of slavery and its relatively recent (last 150 years) dissemination in the Angolan population have thus been suggested [58], [59]. By analogy, as all Brazilian A1 isolates belonged to the ‘Asian-American’, not ‘African’ clade, it is likely that HBV/A1 did not circulate, at least endemically, in western-central Africa, from where originated the great majority of the slaves between the 16^th^ and the 19^th^ century.

As Brazil is the country that received the largest number of slaves coming from different parts of Africa, it is striking that none of the 23 HBV/A1 isolates characterized in this work belonged to the ‘African’ clade. In Haiti, a country whose black population has very similar origins as Brazil, three out of 34 (9%) fully sequenced HBV/A1 isolates belonged to the ‘African’ clade (Fig. 2). The difference between the proportions of ‘African’ isolates in Brazil (0%) and Haiti (9%) was not statistically significant (p>0.2) and may be a result of sampling. In this case, it is possible that members of the ‘African’ clade do circulate in Brazil, at a relatively low rate, and sequencing of a large number of HBV isolates will reveal their presence.

### HBV evolutionary rate

The evolutionary rate of HBV is presently unknown. As mentioned above, a number of studies have proposed evolutionary rates varying from 2.2×10^−6^ to 7.7×10^−4 ^s/s/y, but no consensus has been reached. The tMRCAs for subgenotype A1 and the ‘Asian-American’ clade were calculated by using six different, previously proposed evolutionary rates [38]–[42]. It was deduced that values ranged between 1×10^−5^ and 5×10^−5 ^s/s/y, which translated to tMRCAs of 500–800 and 400–700 years for HBV/A1 and ‘Asian-American clade, respectively, may correspond to the historical reality. In contrast, the extreme values of 2.2×10^−6^ and 7.7×10^−4 ^s/s/y resulted in dating MRCAs at prehistoric times (Mesolithic) and in the 1970s–1980s, respectively (not shown).

In conclusion, the results reported here show that the Brazilian HBV/A1 isolates clustered together with Asian, rather than African isolates, confirming the existence of an ‘Asian-American’ clade within subgenotype A1, and provide insight into the spatial and temporal dynamics of HBV/A1. The close relatedness of the Brazilian, Asian and Somalian isolates suggests that the HBV/A1 strains predominant in Brazil did not originate from the five million slaves, who were imported from Central and Western Africa from 1551 to 1840, but rather from the 300–400,000 captives forcibly removed from southeast Africa at the middle of the 19^th^ century.

## Supporting Information

Table S1 **Nucleotide sequences used in this work.** List of the 151 HBV complete nucleotide sequences (GenBank accession numbers), classified by country, used to construct the phylogenetic tree (Fig. 2). The following criteria were used to include the sequences in the phylogenetic studies: non recombinant human isolates from known country whose nucleotide sequences have been totally determined and did not show any insertion.
(DOCX)
