# Supplementary material for: Hepatitis B Virus Subgenotype A1: Evolutionary Relationships between Brazilian, African and Asian Isolates
Source: PLoS One. 2014 Aug 14;9(8):e105317. doi: 10.1371/journal.pone.0105317 (PMC4133366; doi:10.1371/journal.pone.0105317)
Supplement: Table S1 — Nucleotide sequences used in this work. List of the 151 HBV complete nucleotide sequences (GenBank accession numbers), classified by country, used to construct the phylogenetic tree (Fig. 2). The following criteria were used to include the sequences in the phylogenetic studies: non recombinant human isolates from known country whose nucleotide sequences have been totally determined and did not show any insertion. (DOCX) [file pone.0105317.s001.docx]

**Supplementary material**

**Table S1.** List of the 151 HBV complete nucleotide sequences (GenBank accession numbers), classified by country, used to construct the phylogenetic tree (Fig. 2)

|  |  |  |  |  |  |
| --- | --- | --- | --- | --- | --- |
| Subgenotype A1 | | | | | |
|  | Argentina (n=3) | | | | |
|  | AF043560 | EU185789 | EU366129 |  |  |
|  | Bangladesh (n=2) | | | | |
|  | AB116084 | AB116085 |  |  |  |
|  | Brazil (n=23) | | | | |
|  | KJ854685 | KJ854686 | KJ854687 | KJ854688 | KJ854689 |
|  | KJ854690 | KJ854691 | KJ854692 | KJ854693 | KJ854694 |
|  | KJ854695 | KJ854696 | KJ854697 | KJ854698 | KJ854699 |
|  | KJ854700 | KJ854701 | KJ854702 | KJ854703 | KJ854704 |
|  | KJ854705 | KJ854706 | KJ854707 |  |  |
|  | Colombia (n=4) | | | | |
|  | JQ023660 | JQ023661 | JQ023662 | JQ023663 |  |
|  | Congo (n=1) | | | | |
|  | DQ020002 |  |  |  |  |
|  | France (n=1) | | | | |
|  | AJ309369 |  |  |  |  |
|  | Haiti (n=34) | | | | |
|  | FJ692557 | FJ692558 | FJ692559 | FJ692560 | FJ692561 |
|  | FJ692562 | FJ692563 | FJ692564 | FJ692565 | FJ692566 |
|  | FJ692567 | FJ692568 | FJ692570 | FJ692571 | FJ692572 |
|  | FJ692573 | FJ692574 | FJ692575 | FJ692576 | FJ692577 |
|  | FJ692578 | FJ692579 | FJ692580 | FJ692581 | FJ692582 |
|  | FJ692583 | FJ692584 | FJ692585 | FJ692586 | FJ692587 |
|  | FJ692589 | FJ692590 | FJ692591 | FJ692592 |  |
|  | India (n=7) | | | | |
|  | AB116086 | AB116087 | AY161140 | AY373429 | DQ315784 |
|  | DQ315785 | DQ315786 |  |  |  |
|  | Japan (n=2) | | | | |
|  | AB453986 | AB453988 |  |  |  |
|  | Kenya (n=4) | | | | |
|  | JX154579 | JX154580 | JX154581 | JX154582 |  |
|  | Malawi (n=2) | | | | |
|  | AB076678 | AB076679 |  |  |  |
|  | Martinique (n=6) | | | | |
|  | HE974362 | HE974363 | HE974365 | HE974370 | HE974375 |
|  | HE974381 |  |  |  |  |
|  | Nepal (n=2) | | | | |
|  | AB116088 | AB116089 |  |  |  |
|  | The Philippines (n=7) | | | | |
|  | AB116091 | AB116092 | AB116093 | AB116094 | AY934774 |
|  | EU410082 | M57663 |  |  |  |
|  | Rwanda (n=7) | | | | |
|  | FM199974 | FM199976 | FM199977 | FM199978 | FM199979 |
|  | FM199980 | FM199981 |  |  |  |
|  | Somalia (n=7) | | | | |
|  | AY934765 | AY934766 | AY934767 | AY934768 | AY934769 |
|  | AY934770 | AY934771 |  |  |  |
|  | South Africa (n=18) | | | | |
|  | AF297623 | AY233274 | AY233275 | AY233276 | AY233277 |
|  | AY233278 | AY233279 | AY233281 | AY233282 | AY233283 |
|  | AY233284 | AY233285 | AY233287 | AY233288 | AY233289 |
|  | AY233290 | AY903452 | JN182319 |  |  |
|  | Tanzania (n=1) | | | | |
|  | AY934773 |  |  |  |  |
|  | Uganda (n=1) | | | | |
|  | AY934772 |  |  |  |  |
|  | United Arab Emirates (n=1) | | | | |
|  | DQ020003 |  |  |  |  |
|  | Zimbabwe (n=1) | | | | |
|  | HM535205 |  |  |  |  |
|  |  |  |  |  |  |
| Subgenotype A2 | | | | | |
|  | Brazil (n=3) | | | | |
|  | KJ854708 | KJ854709 | KJ854710 |  |  |
|  | France (n=1) | |  |  |  |
|  | HE576988 |  |  |  |  |
|  | Japan (n=1) | |  |  |  |
|  | AB453979 |  |  |  |  |
|  | South Africa (n=1) | | |  |  |
|  | AY233286 |  |  |  |  |
|  | | | | | |
| Quasi-subgenotype A3 | | | | | |
|  | Gambia (n=2) | |  |  |  |
|  | AY934763 | AY934764 |  |  |  |
|  | Guinea (n=1) | |  |  |  |
|  | GQ161813 |  |  |  |  |
|  | Haiti (n=1) | |  |  |  |
|  | FJ692610 |  |  |  |  |
|  | Mali (n=1) | |  |  |  |
|  | AM180623 |  |  |  |  |
|  | Nigeria (n=3) | |  |  |  |
|  | FJ692554 | FJ692556 | FN545825 |  |  |
|  |  |  |  |  |  |
| Subgenotype A4 | | | | | |
|  | Belgium (n=3) | |  |  |  |
|  | GQ331046 | GQ331047 | GQ331048 |  |  |
|  |  |  |  |  |  |
